# Evaluating diaphragm motor response variability in electric and magnetic phrenic nerve stimulations during passive expiration

**DOI:** 10.1016/j.cnp.2025.11.003

**Published:** 2025-11-25

**Authors:** Ivan Chakalov, Swen Hülsmann, Perianen Ramasawmy, Lukas Diedrich, Mathias Bähr, Leif Saager, Konrad Meissner, Onnen Moerer, Caspar Stephani, Andrea Antal

**Affiliations:** a Department of Anesthesiology, University Medical Center Goettingen, Robert Koch-Strasse 40, 37075 Goettingen, Germany; b Department of Neurology, University Medical Center Goettingen, Robert Koch-Strasse 40, 37075 Goettingen, Germany; c Outcomes Research Consortium, 7000 Fannin Street, Suite 1600, Houston, TX 77030, USA

**Keywords:** Phrenic nerve, CMPNS, CEPNS, TMS, CMAP-Latency, diMEP-Latency

## Abstract

**Objective:**

This study assessed variability in cervical electrical (CEPNS) and magnetic (CMPNS) phrenic nerve stimulation alongside transcranial magnetic stimulation (diTMS) of the diaphragm, focusing on motor response latency and amplitude at individually calculated suprathreshold intensities.

**Methods:**

Diaphragm motor responses were elicited via CEPNS and CMPNS (measuring compound muscle action potentials, CMAPs) and diTMS (measuring diaphragm motor evoked potentials, diMEPs). Latency and amplitude were recorded. Statistical analyses compared methods, evaluated variability using coefficients of variation (CV), and explored associations with height and central motor conduction time.

**Results:**

Among 25 participants (mean age 25 ± 4 years), CMPNS evoked CMAPs in 21 subjects, CEPNS in 16, with 12 responding to both. No significant latency or amplitude differences emerged between CEPNS and CMPNS responders. TMS evoked diMEPs in 24 participants. Latency was more consistent than amplitude across all methods; CMPNS exhibited the lowest amplitude variability. Height moderately correlated positively with CMAP latency and negatively with central motor conduction time.

**Conclusions:**

Individually adapted suprathreshold CEPNS and CMPNS assessed phrenic nerve conduction with varying success rates. Latency was a more reliable measure than amplitude. CMPNS and diTMS surpassed CEPNS in response reliability, positioning magnetic stimulations as preferable for assessing phrenic nerve conduction. CEPNS might not be suitable for examining motor response amplitudes when not applied at supramaximal intensity. Height-related anatomical factors influencing conduction merit further study.

**Significance:**

Latency of diaphragm motor responses elicited at individually estimated stimulation intensities offers a more consistent biomarker over amplitude for assessing phrenic nerve function.

## Highlights

• Variability of electric and magnetic phrenic nerve stimulations.
• Latency is more consistent than amplitude at tailored stimulation intensities.
• Magnetic stimulation methods show higher success than electrical stimulation.

## 1 Introduction

Long-term mechanical ventilation in the intensive care unit (ICU) often leads to diaphragm dysfunction, which is associated with prolonged weaning and poor prognosis (Bithal and Rath, 2024, Jaber et al., 2011, Petrof and Hussain, 2016). Various neurorehabilitation techniques are increasingly applied to critically ill ICU patients with diaphragm dysfunction. These techniques include numerous neuromodulation and neuroplasticity methods aimed at improving respiratory muscle function (Dres et al., 2022, Dubé and Dres, 2016). Transcranial magnetic stimulation of the diaphragm (diTMS) and cervical magnetic phrenic nerve stimulation (CMPNS) are valuable research and clinical tools that can be used to assess diaphragm dysfunction following prolonged mechanical ventilation or to monitor the effects of therapeutic neuromodulation in the ICU. Magnetic stimulation techniques do not require extensive operator training, making them practical and easy to apply in intensive care settings. The combined application of peripheral magnetic and transcranial stimulation enables calculation of central motor conduction time (CMCT)-defined as the latency difference between the central and the peripheral motor response, which also allows differentiation between peripheral and central contributions to diaphragm function (Similowski et al., 1996).

Cervical electrical phrenic nerve stimulation (CEPNS) on the other hand is a well-established gold standard technique with high clinical accuracy (Torrieri et al., 2020). Traditionally, the electrical phrenic nerve stimulation techniques are applied at supramaximal intensities to ensure activation of all motor units by using an intensity clearly above the threshold required for a maximal response, which means the stimulation intensity is not individually adjusted. However, phrenic motor neurons can exhibit structural plasticity in response to altered activation patterns and muscle interactions (Mantilla et al., 2018). Both animal and human studies have demonstrated that peripheral stimuli can induce neuroplasticity changes influencing diaphragm function in various ways (Hopkinson et al., 2012, Popp et al., 2023). Furthermore, noninvasive mechanical ventilation itself reduces the excitability of the diaphragm motor cortex (Sharshar et al., 2004), indicating a negative neuromodulation effect on the corticospinal-diaphragmatic pathway. Therefore, if central or peripheral phrenic nerve stimulations are applied before (baseline measure) and after an phrenic nerve neuromodulation or mechanical ventilation in the ICU to detect such postintervention effects, these changes may be more sensitively identified when the stimulation intensities are individually adjusted based on peripheral and central motor thresholds. Fixed supramaximal intensities without prior threshold determination may obscure or falsely amplify the detection of neuroplasticity-related changes, as response amplitude and latency are influenced by stimulation intensity rather than solely by neural adaptations or modulations (Koseki et al., 2023, Kricheldorff et al., 2022).

Thereby, in this study we compared diaphragm motor responses evoked by cervical electrical (CEPNS), cervical magnetic (CMPNS), and transcranial magnetic stimulation over the diaphragm (diTMS) motor cortex in healthy subjects with the ultimate goal of establishing these stimulation techniques for examining the diaphragm function in the course of mechanical ventilation in the ICU. Unlike in previous studies that applied fixed supramaximal intensities well above individual thresholds for all subjects, our protocol utilized individually estimated stimulation thresholds for all three stimulation modalities, ensuring stimulation intensities were tailored for each test subject. A study from our institution already demonstrated that central transcranial magnetic stimulation of diaphragm (diTMS) can be effectively applied using individually tailored stimulation intensities when biphasic pulses are used instead of monophasic pulses, as biphasic pulses require significantly less charge and time to induce diaphragm motor evoked potentials (diMEPs), allowing for a more efficient and sensitive stimulation protocol (Chakalov et al., 2022). Therefore, to further assess the variability of the two peripheral phrenic nerve stimulation methods applied with individually tailored intensities (CEPNS vs. CMPNS) versus transcranial magnetic stimulation of the diaphragm (diTMS), the coefficients of variation (CVs) of the motor responses from the three methods were compared: compound muscle action potentials (CMAPs) for peripheral stimulations and diaphragm motor evoked potentials (diMEPs) for central stimulation. For the electrical stimulation of the phrenic nerve, it has been shown that the coefficients of variation of the motor responses amplitudes and latencies are under 10 % in primary lateral sclerosis patients (Torrieri et al., 2020). In contrast, other authors observed large within- and between-subject variability in diMEP amplitude following transcranial magnetic stimulation (Welch et al., 2020). Thus, if the application of the three stimulation methods used in our research (CEPNS, CMPNS, and diTMS) demonstrates comparable reliability, only minimal differences in the coefficients of variation for motor response latencies and amplitudes (CMAPs and diMEPs) between methods should be observed, however, substantial interindividual variability in the latency and amplitude of diaphragmatic compound muscle action potentials and motor evoked potentials is expected.

Moreover, we chose to test the three stimulation modalities at the end of passive expiration rather than in the active state (diaphragm contraction during voluntary inspiration). This approach is particularly suitable for patients with diaphragm dysfunction and ventilator weaning failure, who are often unresponsive and for whom supramaximal stimulation in inspiration cannot be applied due to factors such as cardiac rhythm or support devices, intolerance to high stimulation intensities, or the need to evaluate effects of interventions influencing neuroplasticity such as continuous transvenous phrenic nerve neuromodulators (Dres et al., 2022). Hence, such phrenic stimulations respect the limitations of ventilated intensive care patients while still enabling assessment of diaphragm function and neuromodulation effects.

Finally, some previous research suggested that nerve conduction latencies increase with nerve length, which correlates with height (Arulneyam and Manikandan, 2023, Cantone et al., 2023, Puksa et al., 2003). Hence, although the present study was not designed as a biometric analysis, if biometric factors such as height influence latency, these effects should be consistent across methods, allowing height-related latency to serve as an additional measure of variability and consistency among the three phrenic nerve stimulation techniques.

## 2 Materials and methods

### 2.1 Subjects

Twenty-six right-handed subjects aged between 19 and 36 years (mean age 24.84, SD = 4.28) were included in the present study, 25 completed the study, one subject refused to complete the experiment after inclusion. The biometric characteristic of the subjects included are presented on Table 1 and Supplementary Table 1. The responses to electrical phrenic stimulation (CEPNS) of 19 from those 25 subjects were obtained and reported in a previous study (see (Chakalov et al., 2022)). In 14 subjects, CMAPs to cervical magnetic stimulation (CMPNS) of the phrenic nerve were also obtained in randomized order to CEPNS but have not been published so far. Hence, the data of 14 subjects from this previous study (CMAPs to CMPNS, to CEPNS and diMEPS) were analyzed in the context of the present study. Five of those previously tested subjects, whose CMAPs to CMPNS were not obtained in the previous study were tested again in our laboratory using the two methods in randomized order (CMPNS and CEPNS) and additional six subjects were tested in order to achieve a group of 25 subjects, whose diaphragmatic motor responses to CEPNS and CMPNS and TMS were obtained and analyzed for this study.

**Table 1:** Participants biometric data by group and descriptive results.

group gender	height mean (cm)	Height *SD*	Weight mean (kg)	Weight *SD*	BMI mean	BMI *SD*	Age (years)	Age *SD*
***All Subjects***	**171.0**	*9.26*	**69.3**	*9.95*	**23.7**	*2.73*	**24.8**	*4.28*
***Male***	**175.8**	*8.38*	**74.3**	*8.47*	**24.0**	*1.77*	**25.3**	*4.52*
***Female***	**166.7**	*8.04*	**64.7**	*9.18*	**23.3**	*3.43*	**24.4**	*4.14*

Descriptive Data: *Number of Responders per Motor Response- P. Nerve Stimulation Type*		
*All Subjects*	CMAP to CEPNS	*Responders: 16*
*All Subjects*	CMCT- to CEPNS	*Responders: 16*
*All Subjects*	diMEP-diTMS	*Responders: 24*
*All Subjects*	CMAP-CMPNS	*Responders: 21*
*All Subjects*	CMCT-CMPNS	*Responders: 20*
*Male*	CMAP-CEPNS	*Responders: 6*
*Male*	CMCT- CEPNS	*Responders: 6*
*Male*	diMEP-diTMS	*Responders: 11*
*Male*	CMAP-CMPNS	*Responders: 12*
*Male*	CMCT-CMPNS	*Responders: 11*
*Female*	CMAP-CEPNS	*Responders: 10*
*Female*	CMCT-CEPNS	*Responders: 10*
*Female*	diMEP-diTMS	*Responders: 13*
*Female*	CMAP-CMPNS	*Responders: 9*
*Female*	CMCT- CMPNS	*Responders: 9*

Exclusion criteria were previous or ongoing diseases of the central nervous system, implants or metallic material inside the head or neck, cardiac pacemaker, pregnancy, or lactation. The study was approved by the Ethics Committee of the University Medical Center Göttingen (30/1/21) and follows the declaration of Helsinki, including the amendment from Edinburgh (2000). This study was registered as prospective study in the German Clinical Trial Register (reg. number: DRKS Number: 00024449). All subjects gave their informed consent.

### 2.2 Experimental protocol

The cervical magnetic (CMPNS) and electric (CEPNS) stimulations of the phrenic nerve started with defining of the best stimulation coordinates on the neck along the posterior border of the sternocleidomastoid muscle. Before the actual stimulation was conducted the resting motor thresholds were determined for all stimulation modalities used in the present study (i.e., diaphragm motor thresholds at the end of passive expiration). The motor thresholds for both peripheral electric and phrenic nerve stimulations for each subject were individually estimated and the actual stimulations were then applied with suprathreshold intensities similar to the protocol used by these authors (Demoule et al., 2003, Verin et al., 2002). CMPNS and CEPNS were performed in randomized order separated by breaks of 3 to 5 min. The participants sat in a chair equipped with an armrest with their eyes open. The participants’ alertness was constantly monitored by the examiner during the experiment.

After completing the electric and magnetic phrenic nerve stimulations, the experimental protocol for cortical TMS was performed. Diaphragm TMS began with the determination of the hotspot. The search for the hotspot was discontinued if no diMEPs could be evoked within 30 min. According to our previous study that proved the efficacy of different TMS-pulses on the activation of the diaphragm (Chakalov et al., 2022) the cortical magnetic stimulation of the diaphragm was conducted using biphasic pulses that evoke posterior to anterior oriented currents in the brain (anterior-posterior orientation in the coil).

The effectiveness of the three stimulations was judged from the deviation of the real time pattern of the breathing movements registered via ultrasound in B- and M−mode (see Fig. 2) and the absence of concomitant coactivation of the cervical plexus and the long thoracic nerve (innervating the serratus anterior muscle). According to our previous protocol for diaphragm TMS (Chakalov et al., 2022), additional surface electrodes were used to assess co-activation of nearby muscles. Thus, for each of the three stimulation modalities (CEPNS, CMPNS and diTMS) five to fifteen stimulation pulses were delivered at the end of normal expiration with suprathreshold intensity under sonographic control, with at least two respiratory cycles between each stimulation. This approach was used because not every stimulus evoked an effective diaphragm contraction detectable by ultrasound. By applying multiple stimulations, the goal was to obtain at least five effective stimulations for the three methods per individual where the evoked compound muscle action potentials (CMAPs) coincided with observable diaphragm contractions. Hence, stimulation events where the corresponding motor responses were not accompanied by diaphragm contraction, or where signal contamination from non-diaphragmatic muscles was suspected, were considered as contamination from neighboring muscles and were excluded from further analysis to ensure the reliability of the data.

***Cervical Electrical Stimulation (CEPNS) of the Phrenic Nerve:*** CEPNS of the phrenic nerve was performed by means of Digitimer MultiPulse® (model D185-Mark IIa) delivering square-wave pulses of 0.1-ms duration and by using bipolar electrodes placed 2 cm. apart according to already validated previous protocols (see (Demoule et al., 2011, Verin et al., 2002)). the electrodes were placed at the posterior border of the sternocleidomastoid muscles at the level of the cricoid cartilage, 1 to 2 cm. higher or lower, depending on the individual anatomy and motor responses.

For determination of the resting motor threshold for cervical electric stimulation, the Initial stimulations for each subject were performed at an intensity of at least 130 mA (but not exceeding 200 mA), a range previously validated in our laboratory to evoke stable compound muscle action potentials (CMAPs). After identifying the optimal stimulation site, the intensity was gradually decreased until CMAP amplitude disappeared, establishing the threshold level. Subsequently, the stimulation intensity was augmented to above the estimated motor threshold applying an individually estimated suprathreshold intensity that reliably elicited motor responses with stable amplitudes of at least 50 µV. Thus, in contrast to many previous studies, the stimulation intensity was individually adjusted for each subject. Following threshold determination, five to fifteen stimulation pulses were delivered at the end of normal expiration to obtain a comparable number of at least five pulses per method for each individual. Responses without diaphragm contraction or suspected of contamination from neighboring muscles were excluded to ensure data reliability.

***Cervical Magnetic Stimulation (CMPNS):*** The CMPNS was performed using a MagPro X100 device (MagVenture, Dantec S.A., Skovlunde Denmark) with a slightly angulated figure-eight coil (MC-B70).

Because of the specific coil we used, the stimulation technique was slightly modified from previous studies (Similowski et al., 1997, Verin et al., 2002). In our experiment, subjects were asked to turn their heads slightly to the left to facilitate placement of the coil along the right side of the neck so that the center of the coil was approximately over the posterior border of the sternocleidomastoid muscle and the coil handle was cranially oriented (Fig. 2).

In the same way as by the electric stimulation, the cervical magnetic threshold for phrenic nerve stimulations was individually estimated. In all cases CMPNS was performed with biphasic pulses with anterior-posterior orientation in the coil applied at suprathreshold levels. The initial stimulation output power was 36 % of MSO. If this intensity was not sufficient to evoke CMAPs the intensity was gradually increased or decreased by 2 % of MSO until the motor responses appeared. Then the stimulation intensity was gradually decreased by 2 % MSO until the CMAPs amplitude diminished below 50 µV. Finally, the actual stimulation was performed with intensity that is 2 % from MSO above the the last stimulation intensity that still evoked stable CMAPs with amplitude ≥ 50 µV. In the same way as by CEPNS, five to fifteen pulses were delivered for CMPNS at the end of normal expiration (monitored by ultrasound) with at least two breathing cycles in between. Likewise CEPNS, responses without diaphragm contraction or suspected of contamination from neighboring muscles were excluded to ensure data reliability.

***Transcranial Magnetic Stimulation of the Diaphragm (TMS):*** Frameless stereotactically neuronavigated transcranial magnetic stimulation over the cortical representation of the diaphragm (Brainsight TMS Navigation, Rogue Resolutions Ltd coupled with a Polaris-Vicra infrared camera (NDI, Waterloo, Canada)) was performed using the same TMS device and coil as for the CMPNS. The motor hotspot of the diaphragm was determined in a relaxed state (i.e., at the end of passive expiration, Fig. 2) using multistep mapping procedure validated in our laboratory (Chakalov et al., 2022).

The resting motor threshold (RMT) was individually determined using the freely available “Transcranial Magnetic Stimulation Motor Threshold Assessment Tool,” which employs adaptive threshold hunting (Borckardt et al., 2006) based on maximum likelihood estimation (Awiszus, 2003, Awiszus et al., 1999, Groppa et al., 2012). RMT was defined as the TMS intensity that elicited motor-evoked potentials (diMEPs) ≥ 50 µV with > 95 % confidence. Stimulation was applied using 10–15 pulses at 120 %-RMT or 100 % of the maximal stimulator output (MSO) in the cases were the calculated stimulation intensity would exceed the MSO.

***Recording System and Electrode Placement***: Motor responses of the diaphragm to CEPNS, CMPNS (CMAPs), and TMS (MEPs) were recorded using custom silver-chloride surface electrodes (5  mm diameter). After ultrasound assessment of diaphragmatic excursion, electrodes were placed up to 2  cm apart (impedance: 100  Ω–10 kΩ; low-pass filter: 2.5  kHz; sampling: 5  kHz). The active electrode was positioned on the right 7th–9th intercostal space, between the costochondral junction and midclavicular line; the reference electrode was placed slightly lateral on the superior rib, following prior recommendations. (Demoule et al., 2003). EMG signals were amplified using a Digitimer D360 system and digitized via a CED 3001, MICRA 1401 mk II; Cambridge Electronic Design, Cambridge, UK converter. Data were recorded with Signal software (Cambridge Electronic Design, UK).

***Ultrasound:*** B- and M−mode ultrasound measurements were performed using the SonoSite Edge® ultrasound machine (Fujifilm, Sonosite, Inc.) to verify diaphragm activation during both TMS and electrical phrenic stimulation. A curvilinear low-frequency phased-array transducer probe (2,5–5 MHz) was used to register the pattern deviations of the right hemidiaphragm-excursion (contralateral to TMS, Fig. 2). The probe was positioned subcostally at the MCL and angled up- and rightwards, so that the ultrasound beam was projected perpendicular to the right diaphragmatic dome (Boussuges et al., 2009).

## 3 Data analysis and statistics

The latency of the diaphragmatic motor responses (CMAPs or diMEPs) was defined as the time interval from the stimulus onset (induced by CEPNS, CMPNS or TMS) to the beginning of deflection of the EMG signal, while the amplitude was measured as the peak-to-peak voltage difference of the response. A paired *t*-test was then used to compare the latencies and amplitudes of CMAPs evoked by CMPNS and CEPNS.

Secondly, interindividual coefficients of variation (CVs) for diaphragm motor response latencies and amplitudes (CMAPs and diMEPs) were calculated to evaluate the variability of the three stimulation methods: CEPNS, CMPNS, and diTMS. A linear mixed-effects model (LMM) was then fitted to examine the effects of Stimulation Method (CMPNS, CEPNS, diMEP) and Parameter Type (Latency vs. Amplitude) on CVs, including Participant as a random effect to account for repeated measures and missing data. The model was estimated using residual maximum likelihood, assuming a diagonal covariance structure, and model residuals were checked for normality and homoscedasticity. Post-hoc Tukey tests were performed on estimated marginal means for pairwise comparisons where significant main or interaction effects were observed.

**Fig. 1:**
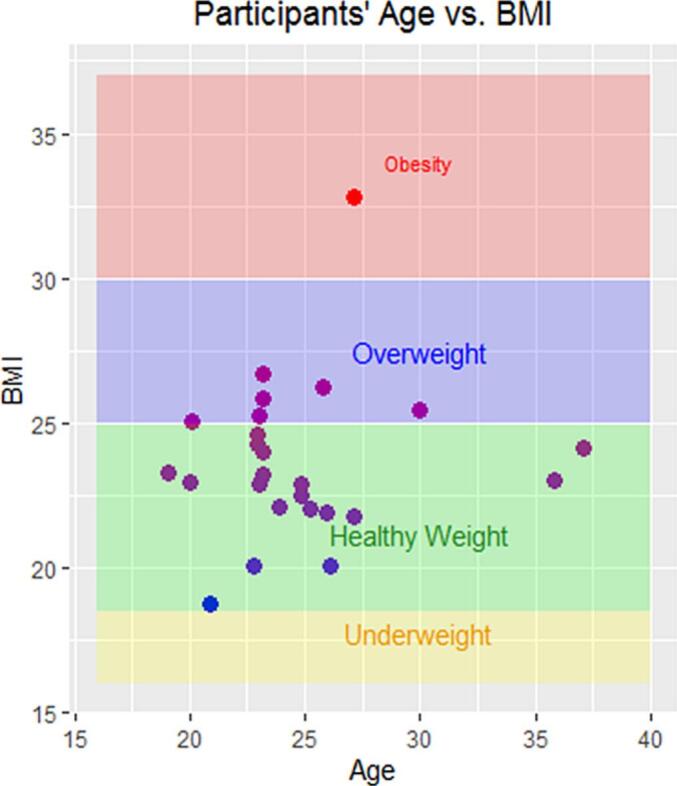
Distribution of Age and BMI of study participants. The figure illustrates that the participants form a relatively homogeneous group, with both age and BMI showing narrow ranges, indicating minimal variation within the cohort.

In addition, if both peripheral stimulation methods were equally reliable, we would expect the resulting CMAP latencies (evoked after CMPNS vs. those evoked after CEPNS) to show a consistent correlation with participants’ height. To test this, Spearman correlation analyses were performed between subject height and CMAP latency after CEPNS and CMPNS, as well as between height and diMEP latency after TMS. We also examined correlations between height and central motor conduction time (calculated as the difference between diMEP and CMAP latencies), and between BMI and motor response latencies for all three stimulation methods. Note that this study was not designed as a biometric investigation. All participants had similar biometric characteristics and age (see Fig. 1). Individual biometric data were used solely as an additional measure to assess the validity of the two phrenic nerve stimulation methods.

Data collection and preprocessing of CMAP/MEP latencies anddiMEPamplitudes followed established procedures (Sommer et al., 2006, Stephani et al., 2016). Data were analyzed using R-Studio (R-Studio, PBC, Boston, MA), and are presented as mean ± standard deviation in the results.

## 4 Results

### 4.1 Descriptive findings

**Fig. 2:**
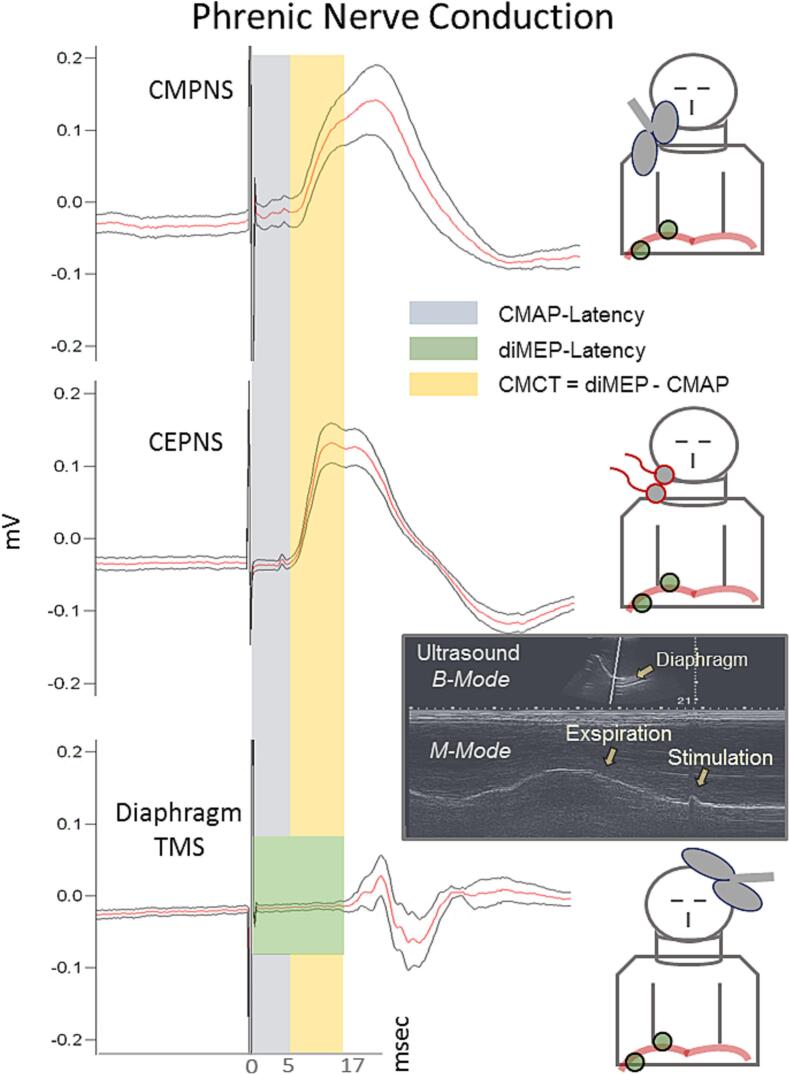
Averaged compound muscle action potentials (CMAPs) and motor evoked potentials (diMEPs) from the diaphragm of one representative test subject. CMAPs and diMEPS are denoted with red lines, with a 95% confidence interval (black contoured) following cervical magnetic stimulation (CMPNS, upper graphic), cervical electric stimulation (CEPNS, in the middle) of the phrenic nerve, and transcranial magnetic stimulation (TMS, bottom graphic) over the cortical representation of the diaphragm. The conduction times and respective latencies are denoted in gray for the CMAPs and green for the MEPs. They are calculated as the time between the appearance of the stimulus artifact and the onset of the electromyographic signal (beginning of the motor response). The central motor conduction time (CMCT) is highlighted in yellow, representing the difference between the latencies of CMAPs after peripheral (CMS and/or CES) and central (TMS) magnetic stimulation of the diaphragm. The effectiveness of the stimulations was monitored in real time via ultrasound-detected deviations of the diaphragm after stimulation.

26 subjects provided a written consent. 25 completed this study (25 ± 4 years of age, 171 ± 9 cm, 69 ± 10 kg, and BMI 25 ± 3). One subject refused to complete the study due to a reflexive syncope that had occurred immediately after the very first CEPNS (Table 1 and Fig. 1). No other severe adverse effects were reported after termination of TMS stimulation. However, neither CEPNS nor CMPNS were able to evoke CMAPs of the diaphragm in all 25 participants. In both cases the attempts to convey CMAPs were terminated after 20 min unsuccessful stimulation and/or if the subjects reported discomfort or pain from the stimulation. Three of the test subjects reported discomfort or pain during attempts to evoke CMAPs with CEPNS. In these cases, the attempts with CEPNS were terminated, and only CMPNS was used to successfully evoke CMAPs. CMAPs were elicited by CMPNS in 21 subjects, whereas only 16 subjects responded to phrenic nerve CEPNS. It is noteworthy that the groups of non-responders (to CEPNS and CMPNS) did not overlap (see Table 2, Table 3). Accordingly, in only 12 from 25 test subjects both methods successfully evoked CMAPs. In all cases where diaphragm motor responses could be evoked, stimulation was accompanied by simultaneous ultrasound registration of diaphragm movements according to a protocol previously established at our institution (see (Chakalov et al., 2022)), see Fig. 2. The number of responders to each phrenic nerve stimulation method and devided by gender are presented on Table 1.

**Table 2:** Latency of the Compound Muscle Action Potentials (CMAP): CEPNS vs CMPNS.

Test subjects	CMAP latency after CEPNS				CMAP latency after CMPNS		
	Latency	SD	CV (%)		Latency	SD	CV (%)
**S01**	Response failure	*	*		8.85	*2.23*	**25.20**
**S02**	*	*	*		8.43	*0.96*	**11.39**
**S03**	*	*	*		6.42	*0.40*	**6.23**
**S04**	4.68	*0.14*	**2.99**		*	*	*
**S05**	3.71	*1.37*	**36.93**		*	*	*
**S06**	*	*	*		5.05	*1.00*	**19.80**
**S07**	4.28	*2.12*	**49.53**		*	*	*
**S08**	4.15	*0.65*	**15.66**		6.62	*0.28*	**4.23**
**S09**	5.60	*0.96*	**17.14**		3.60	*1.23*	**34.17**
**S10**	7.46	*0.43*	**5.76**		6.67	*0.91*	**13.64**
**S11**	8.92	*0.43*	**4.82**		7.31	*1.62*	**22.16**
**S12**	*	*	*		6.96	*0.25*	**3.59**
**S13**	4.38	*0.16*	**3.65**		*	*	*
**S14**	*	*	*		3.01	*0.78*	**25.91**
**S15**	*	*	*		3.01	*0.82*	**27.24**
**S16**	*	*	*		3.64	*0.31*	**8.52**
**S17**	5.48	*0.35*	**6.39**		5.27	*0.79*	**14.99**

**S18**	*Test subject refused to complete the study*						
**S19**	5.52	*0.24*	**4.35**		4.51	*0.90*	**19.96**
**S20**	2.73	*0.23*	**8.42**		2.80	*0.19*	**6.79**
**S21**	4.00	*0.57*	**14.25**		4.40	*0.99*	**22.50**
**S22**	5.82	*0.92*	**15.81**		3.97	*0.96*	**24.18**
**S23**	*	*	*		5.80	*1.74*	**30.00**
**S24**	6.29	*0.12*	**1.91**		4.89	*0.12*	**2.45**
**S25**	8.78	*0.26*	**2.96**		9.03	*0.09*	**1.00**
**S26**	5.18	*0.37*	**7.14**		4.67	*1.92*	**41.1**
**Mean**	**5.44**				**5.47**		
**Betweensubject SD**	***1.74***				***1.92***

**Table 3:** Amplitudes of the diaphragm Compound Muscle Action Potentials (CMAP).

Test subjects	Cervical Elecrical Stimulation (CEPNS)				Cervical Magnetic Stimulation (CMPNS)		
	Individual CMAP amplitude	*Individual- response SD*	CV (%)		Individual CMAP amplitude	*Individual-response SD*	CV (%)
**S01**	*	*	*		0.0103	*0.0020*	**19.42**
**S02**	*	*	*		0.0883	*0.0288*	**32.62**
**S03**	*	*	*		0.0624	*0.0122*	**19.55**
**S04**	0.5999	*0.1997*	**33.29**		*	*	*
**S05**	0.1143	*0.0682*	**59.67**		*	*	*
**S06**	*	*	*		0.0433	*0.0065*	**15.01**
**S07**	0.0950	*0.0565*	**59.47**		*	*	*
**S08**	0.2833	*0.1487*	**52.49**		0.2695	*0.1986*	**73.69**
**S09**	0.1394	*0.0917*	**65.78**		0.1603	*0.0138*	**8.61**
**S10**	0.0381	*0.0043*	**11.29**		0.1554	*0.0475*	**30.57**
**S11**	0.0216	*0.0111*	**51.39**		0.1129	*0.0092*	**8.15**
**S12**	*	*	*		0.0689	*0.0282*	**40.93**
**S13**	0.3110	*0.1075*	**34.57**		*	*	*
**S14**	*	*	*		0.0751	*0.0181*	**24.10**
**S15**	*	*	*		0.0806	*0.0232*	**28.78**
**S16**	*	*	*		0.0409	*0.0133*	**32.52**
**S17**	0.2095	*0.1049*	**50.07**		0.0894	*0.0348*	**38.93**

**S18**	*Test subject refused to complete the study*						
**S19**	0.1964	*0.2035*	**103.62**		0.0320	*0.0097*	**30.31**
**S20**	0.1316	*0.0867*	**65.88**		0.1114	*0.0356*	**31.96**
**S21**	0.0322	*0.0187*	**58.07**		0.1172	*0.0213*	**18.17**
**S22**	0.0347	*0.0086*	**24.78**		0.0635	*0.0218*	**34.33**
**S23**	*	*	*		0.1815	*0.0798*	**43.97**
**S24**	0.1369	*0.0637*	**46.53**		0.1687	*0.0376*	**22.29**
**S25**	0.3082	*0.1499*	**48.64**		0.4357	*0.0411*	**9.43**
**S26**	0.0407	*0.0079*	**19.41**		0.0159	*0.0048*	**30.19**
**Mean**	**0.1683**				**0.1135**		
Betweensubject SD	***0.1515***				***0.0969***

### 4.2 CMAP: CEPNS vs CMPNS

*CMAP-Latency:* The mean CMAP latency for all responders after CEPNS was 5.44 ± 1.74 ms, whereas the mean latency of the CMAP after CMPNS was 5.47 ± 1.92 ms (see Table 2). The mean CMAP latencies from those 12 subjects who responded to both stimulations was 5.83 ± 1.85 ms after CEPNS, and 5.31 ± 1.77 ms after CMPNS. A paired-sample *t*-test indicated that there was no statistically significant difference between the two variables (t = 1.44, df = 11, p = 0.18). The 95 % confidence interval for the mean difference was −0.27 to 1.31, with a sample estimate of 0.52.

*CMAP-Amplitude:* The mean CMAP amplitude for all responders after CEPNS was 0.168 ± 0.152 mV, whereas the mean amplitude of the CMAP after CMPNS was 0.114 ± 0.097 mV (see Table 3). Similarly to latencies, there was no statistically significant difference ((t = 0.51, df = 11, p = 0.62) between the mean CMAP amplitudes after CEPNS (0.131 ± 0.101 mV for those 12 participants who responded to both stimulations) and after CMPNS (0.144 ± 0.114 mV for those 12 participants who responded to both stimulations). The 95 % confidence interval ranged from −0.07 to 0.044.

### 4.3 Diaphragm motor evoked potentials (diMEP) and CMCT

In 24 from 25 subjects who completed the experiment, diMEPs could be elicited by neuronavigated TMS using biphasic pulse configurations (Table 4). The mean stimulation intensity at the RMT was 69.5 ± 16.1 %. The mean diMEP latency for all subjects who responded to TMS stimulation (n = 24) was 16.38 ± 1.50 ms and the respective mean diMEP amplitude 0.225 ± 0.203 mV (Table 4). The mean central motor conduction time (CMCT) of those subjects who responded to one of the two peripheral stimulation methods was 10.71 ± 2.70 after CMPNS (n = 20) and 10.51 ± 2.86 ms after CEPNS (n = 16), respectively.

**Table 4:** Diaphragm Motor Evoked Potentials (diMEP) after TMS.

Test subjects	diMEP-latency				diMEP-amplitude		
	Mean latency	*SD*	CV (%)		Mean amplitude	*SD*	CV (%)
**S01**	20.00	*0.00*	**0.00**		0.1407	*0.0437*	**31.06**
**S02**	15.95	*2.80*	**17.55**		0.0916	*0.0328*	**35.81**
**S03**	17.76	*0.20*	**1.13**		0.2743	*0.0895*	**32.63**
**S04**	15.78	*2.40*	**15.21**		0.1525	*0.0518*	**33.97**
**S05**	17.74	*0.10*	**0.56**		0.1299	*0.0520*	**40.03**
**S06**	16.68	*3.70*	**22.18**		0.2046	*0.0741*	**36.22**
**S07**	17.80	*0.00*	**0.00**		0.8611	*0.3154*	**36.62**
**S08**	16.66	*1.20*	**7.20**		0.2727	*0.2375*	**87.09**
**S09**	17.62	*0.30*	**1.70**		0.0950	*0.0407*	**42.84**
**S10**	13.44	*5.30*	**39.43**		0.1805	*0.1025*	**56.79**
**S11**	13.85	*4.10*	**29.60**		0.4609	*0.2101*	**45.58**
**S12**	15.78	*2.40*	**15.21**		0.0759	*0.0313*	**41.24**
**S13**	15.65	*1.00*	**6.39**		0.6047	*0.2581*	**42.68**
**S14**	17.80	*0.00*	**0.00**		0.0954	*0.0658*	**68.97**
**S15**	*	*	*		*	*	*
**S16**	17.68	*0.20*	**1.13**		0.2506	*0.2825*	**112.73**
**S17**	14.34	*3.80*	**26.50**		0.4771	*0.1482*	**31.06**

**S18**	*Test subject refused to complete the study*						
**S19**	17.28	*1.10*	**6.37**		0.4160	*0.4054*	**97.45**
**S20**	16.55	*2.30*	**13.90**		0.0473	*0.0283*	**59.79**
**S21**	15.44	*2.00*	**12.95**		0.0727	*0.0615*	**84.59**
**S22**	17.14	*1.20*	**7.00**		0.0563	*0.0585*	**103.91**
**S23**	16.40	*0.50*	**3.05**		0.0955	*0.0152*	**15.92**
**S24**	15.64	*2.30*	**14.71**		0.1006	*0.0650*	**64.61**
**S25**	14.4	*3.70*	**25.69**		0.0866	*0.0405*	**46.77**
**S26**	15.78	*2.40*	**15.21**		0.1611	*0.0589*	**36.56**
**Mean**	**16.38**				**0.225**		
Betweensubject SDBetween-subject SD	***1.50***				***0.203***		

### 4.4 Coefficient of variation

The linear mixed effects model revealed significant main effects of Stimulation Method (F(2,116) = 3.86,p = 0.024) and Parameter Type (F(1,116) = 87.01,p < 0.001), as well as a significant interaction between Stimulation Method and Parameter Type (F(2,116) = 9.69,p < 0.001).

Post-hoc estimated marginal means (Tukey-adjusted) showed that amplitude CV differed significantly across stimulation methods. Specifically, CMAP to CEPNS (emmean = 49.1, SE = 4.37) produced higher amplitude variability than CMAP to CMPNS (emmean = 28.3, SE = 3.80, p = 0.007) but did not differ from diMEP to TMS (emmean = 53.5, SE = 3.55, p = 0.97). Across all methods, latency CVs (mean = 13.8, SE = 2.26) were significantly lower than amplitude CVs (mean = 43.6, SE = 2.26; p < 0.001), confirming that latency measures were less variable than amplitude measures (Fig. 4). Among stimulation methods, CMPNS had the lowest average CV (emmean = 22.8, SE = 2.70), followed by CEPNS (emmean = 30.7, SE = 3.11), and diMEP to TMS (emmean = 32.7, SE = 2.51). Pairwise comparisons indicated that CMPNS differed significantly from diMEP (estimate = -9.83, SE = 3.68, p = 0.0238), while differences between CMPNS and CEPNS (estimate = 7.88, SE = 4.12, p = 0.1392) or CEPNS and diMEP (estimate = -1.95, SE = 3.99, p = 0.877) were not significant.

The interaction effects indicated that the difference between amplitude and latency CVs varied by stimulation method. In particular, the amplitude-latency differences were smaller for CMAP to CMPNS compared with CMAP to CEPNS (interaction estimate = 25.8, p = 0.002), while no significant interaction was found involving diMEP to TMS.

### 4.5 Correlation motor response-latency with height and BMI

**Fig. 3:**
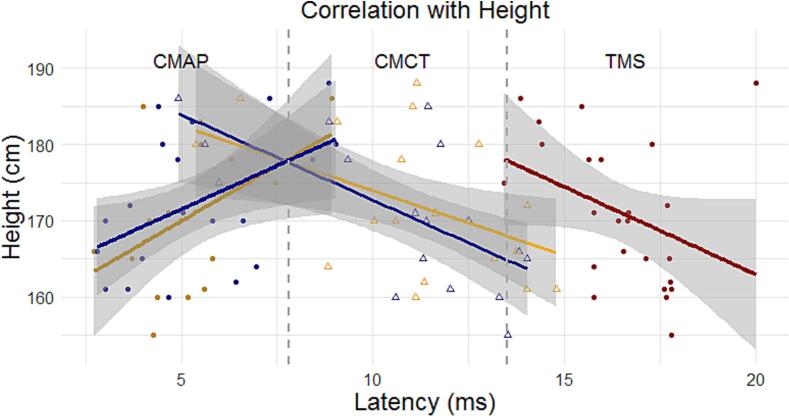
Regression plots, with regression lines and confidence intervals (CI, 95%). Regression plots depict the correlation between latencies of diaphragm CMAPs following CMPNS and CEPNS, as well as diaphragm MEPs after TMS and central motor conduction times (CMCT) respectively, with the height of the test subjects. The regression lines are color-coded: yellow indicates CEPNS, blue indicates CMPNS, and red indicates TMS. The confidence intervals (CIs) are denoted in transparent grey color. The x-axis represents latencies in milliseconds (ms), while the y-axis represents the height of the subjects in centimeters (cm).

The Spearman correlation between CMAP latency after CEPNS and *Height* showed positive moderate correlation (r = 0.38) but couldn’t reach statistical significance (p = 0.14), based on 16 valid observations. The positive correlation between CMAP latency after CMPNS and *Height* was moderate (r = 0.47) and statistically significant (p = 0.03), based on 21 valid observations (see Fig. 3 and Suppl Table 2).

**Fig. 4:**
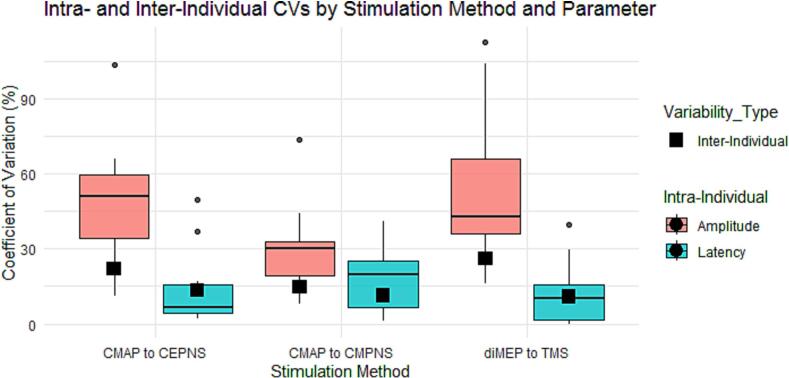
Boxplots with error bars denoting the comparison of the Coefficients of Variation (CV, %) of Latencies (blue boxes) and Amplitudes (red boxes) of diaphragm muscle action potentials (CMAPs) and motor evoked potentials (MEPs). The boxplots represent the intra-individual variability (large boxes) and the inter-individual variability (small black boxes) of the motor responses to electrical and magnetic stimulations of the phrenic nerve. The CMAPs and MEPs were recorded after cervical electrical stimulation (CEPNS), cervical magnetic stimulation (CMPNS), and transcranial magnetic stimulation (TMS) over the diaphragm representation. The boxplots show that the diaphragm motor response latencies evoked at individually estimated suprathreshold intensities are significantly less variable than the amplitudes for all stimulation modalities. Additionally, the intra-individual responses appear to have greater amplitude variability than the inter-individual responses.

Spearman correlation between participants' *Height* and the Central Motor Conduction Time (CMCT) after cervical phrenic nerve electric and magnetic stimulations showed a significant negative moderate to strong correlation in both cases, CMCT-CMPNS (r = − 0.47, p = 0.04, df = 18) CMCT-CEPNS (r = − 0.63, p = 0.01, df = 14), Fig. 3 and Suppl Table 2. The latencies of the diaphragm motor evoked potentials (diMEPs) after cortical TMS also demonstrated a moderate negative correlation with height, that didn’t reach significance (r = -0.39, p = 0.06, df = 22). Additionally, Supplementary Table 2 represents the correlations between height and latency stratified by gender.

In contrast, no significant correlations were observed between BMI and motor responses, nor between the weight of the subjects and motor responses, regardless of the stimulation method. However, a significant moderate positive correlation was detected between diMEPs and BMI (r = 0.50, p = 0.02, df = 22) In contrast, the corresponding correlation analyses between BMI and CMAP −CEPNS (r = − 0.07, p = 0.79, n = 14), CMAP-CMPNS (r = -0.20, p = 0.39, n = 19) as well as CMCT-CEPNS (r = − 0.31, p = 0.25, df = 14) and CMCT-CMPNS (r = 0.39, p = 0.86, df = 18) were all negative and statistically not significant.

## 5 Discussion

The main goal of the present study was to evaluate the motor response variability of three phrenic nerve stimulation techniques; cervical electrical (CEPNS), cervical magnetic (CMPNS), and transcranial magnetic stimulation of the diaphragm (diTMS), using individually tailored stimulation intensities, rather than traditional fixed supramaximal levels. In our study the response rates differed across modalities, the peripheral electrical phrenic nerve stimulation demonstrated the highest rate of response failure compared to cervical magnetic stimulation and transcranial magnetic stimulation of the diaphragm. For peripheral phrenic stimulations, 21 out of 25 subjects responded to CMPNS, while 16 out of 25 responded to CEPNS; notably, CEPNS was successful in 4 cases where CMPNS failed, and CMPNS in 9 cases where CEPNS did not. This does not definitively establish magnetic phrenic nerve stimulation techniques as superior, as factors such as discomfort during CEPNS, the use of individually adapted stimulation intensities and square-wave pulses may have reduced its efficacy (Collu et al., 2023, Gracanin and Trnkoczy, 1975). CEPNS is well established technique and remains the clinical gold standard for accuracy (Durand et al., 2005, Jenkins et al., 2016, Maranhão et al., 2017, Pinto et al., 2012, Pinto et al., 2009, Silva et al., 2020), but CMPNS is generally more practical and less time-consuming, particularly for supine ICU patients, because it provides nonvolitional test of diaphragm strength (Wragg et al., 1994). With growing interest in phrenic nerve neuromodulation techniques for critically ill ICU-patients (Dres et al., 2022, Reynolds et al., 2017), reliable methods for assessing diaphragm function are essential for monitoring both central and peripheral neuroplasticity. All three stimulation modalities were applied at the end of passive expiration, reflecting real-world ICU constraints, where supramaximal intensities and voluntary inspiration may be unfeasible due to patient sedation, intolerance, or support device interference. Previous studies have demonstrated that diaphragmatic activity persists during expiration even in disease conditions, and that testing at the end of expiration can provide relevant information on neuromechanical coupling and preservation of lung volume (Emeriaud et al., 2006, Pellegrini et al., 2017). Submaximal stimulation also allowed exploration of variability in latency vs. amplitude as clinically relevant markers, since maximal intensities were avoided for comfort and safety.

Our analysis of coefficients of variation (CVs) showed that amplitude measurements were consistently more variable than latency, confirming latency as the more reliable marker of phrenic nerve conduction. Among techniques, CMPNS produced the most stable amplitudes, outperforming CEPNS and diTMS, though latency variability did not differ significantly between methods. A few subjects showed higher CMAP-latency variability, likely due to contamination from neighboring muscles. Overall, CMPNS appears preferable when amplitude is of interest, while latency measures are comparably robust across all stimulation approaches. Importantly, the expected variability in the diaphragm motor evoked potential (diMEP) amplitude was observed, consistent with literature findings of decreased amplitude variability at higher stimulus intensities and muscle contraction levels (Darling et al., 2006). However, fixed supramaximal stimulation, as traditionally used in clinical protocols, does not account for individual differences in motor threshold and may mask subtle neuroplasticity changes-an important consideration when assessing post-intervention effects or effects of mechanical ventilation in ICU patients.

Bipolar electrodes for cervical electric phrenic nerve stimulation are typically positioned on the skin over the cervical region of the neck, usually along the anterior or lateral aspects near the sternocleidomastoid muscles (Similowski et al., 1997, Verin et al., 2002). In contrast, for cervical magnetic stimulation, the coil is typically positioned parallel to the spine or at a 45° angle, with the coil handle directed either caudally or cranially (Similowski et al., 1997, Verin et al., 2002). In this setup, as in our study, the electrodes of the CEPNS are closer to the origin of the phrenic nerve, allowing more precise control of the stimulation compared to CMPNS. CMPNS, on the other hand, is easier to apply and takes less time, because it does not require precise positioning of the coil. This feature may be particularly advantageous in certain patient populations, as the anterolateral positioning of the coil facilitates the use of magnetic stimulation in supine patients, such as those in the operating room or intensive care unit (Gary H Mills et al., 1995). Therefore, the success of the stimulation depends on the specific subject and context of stimulation. The overall lower variability of latencies compared to amplitudes in motor evoked responses is consistent with previous experience from transcranial magnetic stimulation (TMS) research on other skeletal muscles (Ammann et al., 2020, Darling et al., 2006, Hashemirad et al., 2017, Pellegrini et al., 2018, Schilberg et al., 2021). A recent study suggests that cortical alpha and beta oscillations may modulate the amplitudes and contribute to the high variability of motor-evoked potentials by influencing corticospinal excitability. (Schilberg et al., 2021). In addition, the number of trials and the number of subjects being tested can also significantly influence the MEP amplitudes (Ammann et al., 2020). In the specific context of phrenic nerve stimulation, our findings are supported by previous work (Laghi et al., 1996), which demonstrated that magnetic stimulation of the phrenic nerves leads to consistent diaphragm recruitment. This consistency may account for the lower variability in CMAP latencies observed following cervical electric and magnetic phrenic nerve stimulation.

As an additional check of variability among the three phrenic nerve stimulation methods, motor response latencies (CMAPs and diMEPs) were compared in relation to body height across stimulation methods. Note that the present study was not designed as a biometric study; height was used solely as an additional tool to assess variability and, consequently, reliability of the three methods. Both CEPNS and CMPNS showed a moderate positive correlation with height, reaching significance only for CMPNS, likely due to its larger responder group. This finding aligns with established evidence that nerve conduction latencies increase with height, i.e. taller individuals tend to have longer latencies (Cantone et al., 2023, Tobimatsu et al., 2019). No significant correlations were observed between BMI and CMAP latencies for either method. Additionally, height was inversely correlated with central motor conduction time (CMCT) to the diaphragm for both CEPNS and CMPNS, with a stronger negative correlation after CEPNS. Previous studies have shown mixed results regarding height and CMCT in different muscles (Imajo et al., 2017), with some reporting no correlation for diaphragmatic CMCT (Ansari and Hahn, 2017). Further biometric research involving larger and more diverse populations is needed to clarify the influence of height and other physical factors on diaphragm neural conduction.

A primary limitation of this study is that not all subjects responded to every stimulation method, which likely reflects the fact that stimulations were performed at individually determined suprathreshold intensities, rather than at supramaximal intensities. Stimulation dose safety in TMS varies between individuals, and acceptable risk levels differ depending on whether the individual receiving stimulation belongs to a vulnerable population (Rossi et al., 2021). Individually estimated suprathreshold stimulation ensures subject comfort and safety, especially concerning TMS stimulations, but may not fully account for inter-individual differences in nerve excitability and muscle response thresholds, resulting in a proportion of non-responders for each technique. This variability aligns with existing literature reporting that electrode positioning, subject anatomy, and precise titration of stimulation intensity play a critical role in successful phrenic nerve and diaphragm activation, and that submaximal stimulation may result in inconsistent capture of motor responses across (Keogh et al., 2022). Unlike the two magnetic stimulations (peripheral and central), which reliably evoked diaphragm motor responses in most test subjects, the cervical electric phrenic nerve stimulation showed not only a very high rate of response failure but also a high vulnerability of motor response amplitudes when executed at individually adjusted suprathreshold (non-supramaximal) intensity. The cervical electrical stimulation (CEPNS) applied at supramaximal intensity remains the gold standard technique for diagnosis of phrenic nerve conduction with high clinical accuracy (Torrieri et al., 2020) and using alternative electrode montages in the clinic may reduce the failure rates (e.g., (Maranhão et al., 2017)). However, the electrical stimulation of the phrenic nerve at individually adjusted submaximal intensities is technically challenging and probably less appropriate for detections of neuroplasticity-based amplitude changes of diaphragm motor responses than magnetic stimulations.

Furthermore, the present study used a homogeneous and relatively small sample of young adults, and stimulation intensities were limited to levels tolerable for participants. As a result, some responses may have been missed due to insufficient stimulation, despite careful threshold estimation and sonographic confirmation of diaphragm contraction. This methodological constraint may limit the generalizability of our findings, particularly regarding the efficacy and reliability of the cervical electric stimulation in more diverse clinical populations, such as older adults or patients with nerve or muscle impairment. However, since most ICU patients are under sedation and analgesia, the use of higher stimulation intensities in such settings would likely reduce the failure rate (Boutros et al., 2025, Dres et al., 2022).

Future studies should explore the use of adaptive thresholding and closed-loop systems to maximize response rates without compromising participant safety. Trials involving ventilated patients and improved monitoring of individual stimulation responses, such as employing a esophagus NAVA probe (neurally adjusted ventilatory assist), which is highly sensitive to even minimal differences in the electromyographic signal (Shah et al., 2024), as well as new-generation ventilators capable of detecting milliliter-level changes in lung volume and diaphragm movement, could clarify the benefits of individually adapted magnetic and electric phrenic nerve stimulation intensity in clinical settings.

## 6 Conclusion

The present study demonstrates that cervical electrical and magnetic phrenic nerve stimulation applied with individually adapted suprathreshold intensities reliably assess phrenic nerve conduction, with latency showing greater consistency than amplitude across methods. Height correlated with CMAP latency and central motor conduction time, suggesting anatomical influences worth further study. Latency measures appear robust for clinical and research use, while method selection should consider patient characteristics and context. The electrical phrenic nerve stimulation demonstrated the highest rate of response failure compared to cervical magnetic stimulation and transcranial magnetic stimulation of the diaphragm, making diTMS a superior technique for assessing phrenic nerve conduction (peripheral or central), particularly when stimulations are not applied at supramaximal intensities, but individually estimated suprathreshold intensities. Further research, especially in heterogenic ventilated patients using improved monitoring methods such as highly sensitive neurally adjusted ventilatory assist is warranted to examine the stimulation success rates of the three methods in pathological state.

Authorship contribution statement

I.C. collected data, I.C. and P.R. performed data analysis, all co-authors participated in writing the manuscript and controlling the data analysis. I.C. prepared figures and tables and accompanied the study as a physician. A.A., S.H., M.B., O.M., L.D., L.S., K.M. and C.S. participated in the study designed and supervised the data collection and analysis and reviewed the manuscript.

## Ethics approval statement

This study was approved by the ethics committee of the University of Göttingen (ethic committee number 30/1/21) and conforms to the Declaration of Helsinki.

## Declaration of competing interest

The authors declare the following financial interests/personal relationships which may be considered as potential competing interests: IC was promoted by “German Research Foundation-Clinician Scientist” academic qualification program (DFG-CS) and received financial support to perform the experiments in parallel to his specialist training in the University Medical Center in Göttingen, project number: 413501650. The other authors declare that they have no known competing financial interests or personal relationships that could have appeared to influence the work reported in this paper.
