# Supplementary material for: Evaluating diaphragm motor response variability in electric and magnetic phrenic nerve stimulations during passive expiration
Source: Clin Neurophysiol Pract. 2025 Nov 25;10:529–39. doi: 10.1016/j.cnp.2025.11.003 (PMC12702034; doi:10.1016/j.cnp.2025.11.003)
Supplement: Supplementary Data 1 [file mmc1.docx]

| **Supplementary Table 1. Participants’ biometric data** | | | | | |
| --- | --- | --- | --- | --- | --- |
| **Test Subjects** | **Height** | **Weight** | **BMI** | **Age** | **Gender** |
| **S01** | **188** | 90 | **25.46** | *30* | **m** |
| **S02** | **178** | 73 | **23.04** | *36* | **m** |
| **S03** | **162** | 61 | **23.24** | *23* | **w** |
| **S04** | **171** | 68 | **23.26** | *19* | **w** |
| **S05** | **165** | 66 | **24.24** | *23* | **w** |
| **S06** | **171** | 78 | **26.67** | *23* | **m** |
| **S07** | **155** | 54 | **22.48** | *25* | **w** |
| **S08** | **170** | 72.5 | **25.09** | *20* | **w** |
| **S09** | **161** | 85 | **32.79** | *27* | **w** |
| **S10** | **175** | 70 | **22.86** | *25* | **m** |
| **S11** | **186** | 83 | **23.99** | *23* | **m** |
| **S12** | **164** | 65 | **24.17** | *37* | **w** |
| **S13** | **160** | 48 | **18.75** | *21* | **w** |
| **S14** | **161** | 67 | **25.85** | *23* | **m** |
| **S15** | **170** | 73 | **25.26** | *23* | **m** |
| **S16** | **172** | 68 | **22.99** | *20* | **m** |
| **S17** | **183** | 73 | **21.8** | *27* | **w** |
| **S18** | *Test subject refused to complete the study* | | | | **m** |
| **S19** | **180** | 85 | **26.23** | *26* | **m** |
| **S20** | **166** | 63 | **22.86** | *23* | **w** |
| **S21** | **185** | 75 | **21.91** | *26* | **m** |
| **S22** | **165** | 60 | **22.04** | *25* | **m** |
| **S23** | **170** | 58 | **20.07** | *23* | **w** |
| **S24** | **178** | 70 | **22.09** | *24* | **m** |
| **S25** | **180** | 65 | **20.06** | *26* | **w** |
| **S26** | **160** | 63 | **24.61** | *23* | **w** |
| ***Mean*** | **171.04** | **69.34** | **23.6724** | ***24.84*** |  |
| ***Between Subjects SD*** | **9.2622** | **9.9506** | **2.7285** | **4.2786** |  |
