# Supplementary material for: Evaluating diaphragm motor response variability in electric and magnetic phrenic nerve stimulations during passive expiration
Source: Clin Neurophysiol Pract. 2025 Nov 25;10:529–39. doi: 10.1016/j.cnp.2025.11.003 (PMC12702034; doi:10.1016/j.cnp.2025.11.003)
Supplement: Supplementary Data 2 [file mmc2.docx]

| **Suppl-Table 2: Correlation of Height and Motor Response Latency by Gender/Group** | | | | | | | |
| --- | --- | --- | --- | --- | --- | --- | --- |
| **Gender/ Group** | **PN Stimulation**  **Method** | **Motor Response Mean**  **(ms)** | **Motor Response Mean**  ***SD*** | **Height Mean (cm)** | **Spearman**  **r-value** | ***p-*value** | ***N*** |
| ***All***  ***Subjects*** | CMAP-CEPNS | **5.44** | *1.74* | **171.0** | **0.382** | *0.145* | **16** |
| ***All***  ***Subjects*** | CMCT- CEPNS | **10.63** | *2.94* | **171.0** | **-0.626** | *0.009******** | **16** |
| ***All***  ***Subjects*** | diMEP | **16.38** | *1.50* | **171.0** | **-0.396** | *0.055* | **24** |
| ***All***  ***Subjects*** | CMAP-CMPNS | **5.47** | *1.92* | **171.0** | **0.468** | *0.032******** | **21** |
| ***All***  ***Subjects*** | CMCT-CMPNS | **10.71** | *2.70* | **171.0** | **-0.468** | *0.037******** | **20** |
| **Male** | CMAP-CEPNS | **6.34** | *1.69* | **175.8** | **0.029** | *0.957* | **6** |
| **Male** | CMCT- CEPNS | **9.13** | *2.99* | **175.8** | **-0.086** | *0.872* | **6** |
| **Male** | diMEP | **16.45** | *1.87* | **175.8** | **-0.205** | *0.545* | **11** |
| **Male** | CMAP-CMPNS | **5.31** | *2.02* | **175.8** | **0.709** | *0.010******** | **12** |
| **Male** | CMCT-CMPNS | **10.92** | *2.86* | **175.8** | **-0.597** | *0.053* | **11** |
| ***Female*** | CMAP-CEPNS | **5.44** | *1.74* | **166.7** | **0.274** | *0.444* | **10** |
| ***Female*** | CMCT-CEPNS | **10.63** | *2.94* | **166.7** | **-0.517** | *0.126* | **10** |
| ***Female*** | diMEP | **16.38** | *1.50* | **166.7** | **-0.493** | *0.087* | **13** |
| ***Female*** | CMAP-CMPNS | **5.47** | *1.92* | **166.7** | **0.385** | *0.306* | **9** |
| ***Female*** | CMCT- CMPNS | **10.71** | *2.70* | **166.7** | **-0.644** | *0.061* | **9** |
